# Natural cannabinoids effects on glutamatergic and dopaminergic neurotransmission in a transgenic model of Alzheimer's disease

**DOI:** 10.1002/alz70855_102846

**Published:** 2025-12-26

**Authors:** Paula Subirana, Núria Sánchez, Francisco Ciruela, Jordi Bonaventura, Ester Aso

**Affiliations:** ^1^ Universitat de Barcelona, Barcelona, Barcelona, Spain

## Abstract

**Background:**

Previous results demonstrated that chronic treatment with a combination of two natural cannabinoids, Δ^9^‐tetrahydrocannabinol (THC) and cannabidiol (CBD), at non‐psychotropic doses reduces cognitive decline, as well as the extracellular glutamate levels and the basal excitability in the hippocampus of APP/PS1 mice. In the present study, we aimed to elucidate whether this modulation of hippocampal excitability exerted by natural cannabinoids could affect the dopaminergic activity in limbic areas related to non‐cognitive symptoms of Alzheimer's disease (AD) in our animal model.

**Method:**

We used glutamate and dopamine biosensors, along with fiber photometry techniques, to evaluate the levels of these neurotransmitters in the hippocampus and nucleus accumbens (NAcc), respectively. Experiments were conducted in anaesthetized animals for recording under an electrical hippocampal stimulation protocol, or in awake animals for recording during behavioral evaluations (novel object recognition, open field, sociability and prepulse inhibition tests).

**Result:**

Chronic treatment with THC and CBD reversed the increased prominence and frequency of glutamate peaks observed in the hippocampus of APP/PS1 animals during the novel object recognition test at early stages of the AD‐like process. At more advanced stages, APP/PS1 mice exhibited alterations in dopamine dynamics in the NAcc, which were compatible with psychotic‐like traits observed in this animal model of AD. Interestingly, these alterations were partially modulated by chronic treatment with these natural cannabinoids.

**Conclusion:**

Our results reveal that the combination of THC and CBD modulates glutamatergic activity in the hippocampus at early stages of the AD process and that, likely related to this, reduces dopaminergic alterations in limbic areas at advanced stages. Thus, these natural cannabinoids may alleviate both cognitive and non‐cognitive symptoms occurring in AD, supporting their clinical development as a pleiotropic therapeutic alternative for this neurodegenerative disease.

